# Correction to “Upper Versus Middle‐Neck Involvement in TNM‐9 Stage IB Nasopharyngeal Carcinoma: Implications for De‐Escalation and Intensification Strategies”

**DOI:** 10.1002/cam4.72299

**Published:** 2026-09-14

H. Xu , X. Chen , D. Huang , et al., “Upper Versus Middle‐Neck Involvement in TNM‐9 Stage IB Nasopharyngeal Carcinoma: Implications for De‐Escalation and Intensification Strategies,” Cancer Medicine 15, no. 8 (2026): e72166, 10.1002/cam4.72166.42557921 PMC13444347

In the Ethics Statement section, the ethics approval number “K2024‐029‐01” was incorrect. The updated Ethics Statement section is as follows:

## Ethics Statement

This retrospective study was approved by the ethics committee of Fujian Cancer Hospital (Approval no. K2026‐039‐01). The requirement for written informed consent was waived due to the retrospective nature of the study.

We apologize for this error.

